# The role of lncRNAs and *XIST* in oral cancer

**DOI:** 10.3389/fcell.2022.826650

**Published:** 2022-08-10

**Authors:** Huimin Liu, Dongxu Wang, Shaoning Kan, Ming Hao, Lu Chang, Pengxu Lu, Yangyang Liu, Ye Jin, Weiwei Liu

**Affiliations:** ^1^ Department of Oral and Maxillofacial Surgery, Hospital of Stomatology, Jilin University, Changchun, China; ^2^ Laboratory Animal Center, College of Animal Science, Jilin University, Changchun, China; ^3^ School of Pharmacy, Changchun University of Chinese Medicine, Changchun, China; ^4^ Jilin Provincial Key Laboratory of Tooth Development and Bone Remodeling, Hospital of Stomatology, Jilin University, Changchun, China

**Keywords:** gene expression, long non-coding RNA, oral cancer, pathogenesis, *XIST*

## Abstract

Long non-coding RNA (lncRNA) plays a significant role in the pathogenesis of many human malignant tumors, including oral cancer. LncRNA can act as a gene regulator in a variety of cancers. It regulates the growth of malignant cells *via* many cellular signal pathways such as the PI3K (phosphoinositide 3-kinase)/AKT (α-serine/threonine-protein kinase) pathway. In this review, we have analyzed the role of lncRNAs, such as lncRNA X inactive specific transcript (*XIST*), in oral cancer, including its effects on the proliferation, apoptosis, invasion, migration, and resistance to chemotherapy of oral cancer. We have also focused on the role of lncRNA *XIST* as the core of X chromosome inactivation. Here, we provide a brief overview of the role of many kinds of lncRNAs, including *XIST*, which provides a theoretical basis for the study of the role of *XIST* in oral cancer. Our review may provide a new direction for the study of the occurrence, development, and prognosis of oral cancer and provide a new target for its treatment.

## Introduction

Oral cancer is the 11th most common carcinoma around the globe that has attracted global attention (D'Souza and Addepalli, 2018). There were 377,713 new cases and 177,757 new deaths of oral cancer in 2020 (Sung et al., 2021). Most patients are diagnosed with oral cancer at an advanced stage (Bagan et al., 2010). It is characterized by a poor prognosis and a high rate of lymphatic metastasis (Okura et al., 2009; Noguti et al., 2012). Surgical resection in combination with radiotherapy and chemotherapy is currently the main treatment for oral cancer. However, this treatment regimen has not helped in significantly improving the five-year survival rate (Alkhadar et al., 2021). There is mounting evidence that has demonstrated that lncRNAs play a crucial role related to the survival rate in oral cancer (Zhang et al., 2018a; Momen-Heravi and Bala, 2018; Gao et al., 2020; Chen et al., 2021). Moreover, the expression pattern of lncRNAs plays a role in the diagnosis and therapy of oral cancer (Momen-Heravi and Bala, 2018). LncRNA *MALAT1* can be used as a biomarker and therapeutic target for oral squamous cell carcinoma (OSCC) (Zhou et al., 2015).

**FIGURE 1:**
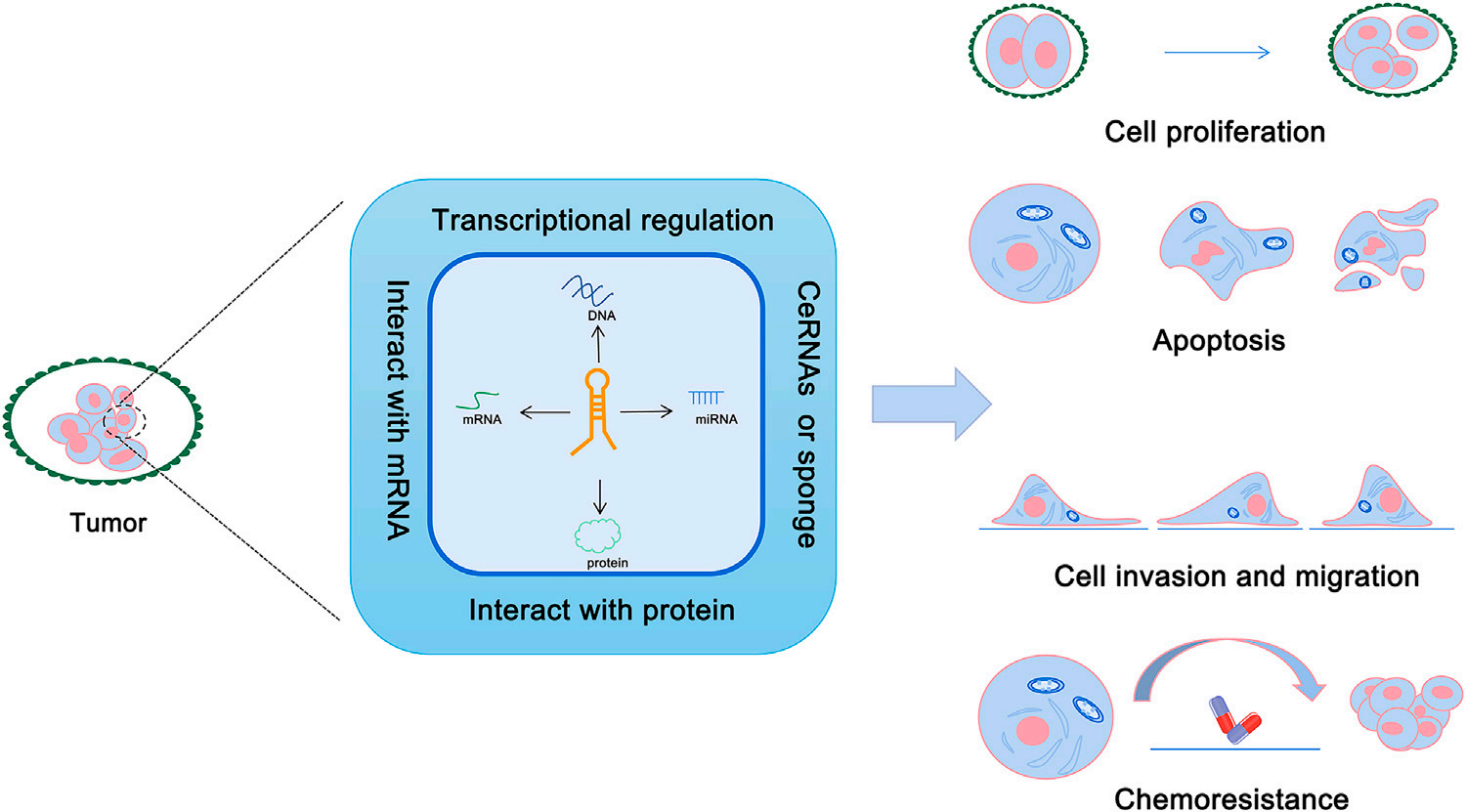
Roles of lncRNAs in cancer.

With the in-depth study of the human genome, it has been found that most of the genome can be transcribed into RNA, yet only 1%–2% of the protein-coding genes encode proteins involved in various cellular life activities (Beermann et al., 2016). Compare to the coding RNAs, non-coding RNAs were initially considered to be the “junk DNA,” which were produced during the process of gene transcription (Doolittle, 2013). However, it has been found that non-coding RNA plays a critical regulatory role in gene expression and other biological processes (Anastasiadou et al., 2018). Non-coding RNAs include microRNAs, circRNAs, intron RNAs, and long non-coding RNAs (Morris and Mattick, 2014). Long non-coding RNAs (lncRNAs) are defined as any non-protein-coding RNA >200 bp in length (Morris and Mattick, 2014; Qian et al., 2019). LncRNAs help to protect the integrity of the genome and regulate gene expression by interacting with DNA, RNA, and proteins (Mercer et al., 2009; Chen, 2016). Moreover, lncRNA can play multiple roles in pathogenesis of tumors *via* different mechanisms (Figure 1). A recent study has shown that lncRNA *XIST* is one of the lncRNAs that play a key role in the pathogenesis of OSCC (Tao et al., 2021). Results from this study demonstrated that *XIST* inhibited apoptosis, and promoted cell proliferation, invasion, and migration to promote the growth of oral cancer (Tao et al., 2021). The findings of this study provide a new direction for the study of *XIST* in oral cancer.

## The role of lncRNA in oral cancer

Studies have shown that there is a variety of abnormal expression patterns of lncRNAs in oral cancer, and they have a critical role in the pathogenesis of cancer (Momen-Heravi and Bala, 2018). Different lncRNAs have a variety of different effects on oral cancer (Fang et al., 2020; Ghafouri-Fard et al., 2020; Sur et al., 2020). *H19*, the first lncRNA to be discovered, is considered to be closely related to embryonic development and tumorigenesis (Raveh et al., 2015; Zhang et al., 2017; Wang et al., 2019a; Kou et al., 2019; Zhang et al., 2020). *H19* is abnormally expressed in oral cancer and plays a role as a “sponge molecule” (Kou et al., 2019). It regulates the expression of the high mobility group A2 (HMGA2) protein through miRNA let-7 to promote the migration and invasion of tongue squamous cell carcinoma (TSCC) (Kou et al., 2019). In addition to *H19*, there are many lncRNAs that aggravate the development of oral cancer by promoting the migration and invasion of cancer cells, such as lncRNA *FGD5-AS1* (Liu et al., 2020a), lncRNA *MYOSLID* (Xiong et al., 2019), and lncRNA *HOTAIR* (Tao et al., 2020). In addition, lncRNAs such as lncRNA *CASC9* can also promote the growth of cancerous cells by suppressing autophagy-mediated cell apoptosis in oral cancer (Yang et al., 2019). It has been reported previously that lncRNA *CASC9* plays a vital role in the pathogenesis of OSCC and inhibits apoptosis and autophagy *via* the AKT (Protein kinase B)/mTOR (mammalian target of rapamycin) signal pathway (Yang et al., 2019). LncRNA *HIFCAR* can be used as a co-activating factor of hypoxia-inducible factor HIF-1α for regulating the hypoxia signal pathway in oral cancer (Shih et al., 2017). It also plays a role in the pathogenesis of oral cancer, thereby suggesting that it could be a new therapeutic target for the treatment of oral cancer (Shih et al., 2017). It has also been reported that lncRNA *HAS2-AS1* was abnormally expressed in oral cancer, which was closely related to the anoxic state of oral cancer (Zhu et al., 2017).

Resistance to chemotherapeutic drugs is a major challenge in the treatment of cancer (Cui et al., 2018; Bukowski et al., 2020). One of the key research focus areas in oncology research is to identify the mechanisms by which resistance is developed by the cancer cells to chemotherapeutic drugs (Li et al., 2020a). Studies have found that lncRNA *UCA1* was highly expressed in cisplatin-resistant OSCC (Fang et al., 2017). There is also mounting evidence that lncRNA *UCA1* can promote the proliferation of OSCC cells and inhibit the sensitivity of OSCC cells to cisplatin (Fang et al., 2017). Meanwhile, the aforementioned results suggested that lncRNA *UCA1* also can regulate the growth of OSCC *via* miR-184 (Fang et al., 2017). This suggested that an interaction between lncRNA and miRNA might be able to regulate the pathogenesis of oral cancer (Zhou et al., 2019).

*XIST* is an lncRNA located in the *XIST* gene, which plays a key role in dose compensation of the X chromosome and embryonic development (Sahakyan et al., 2018). As a key regulator of chromosome dose compensation, *XIST* can achieve dose compensation by randomly inactivating the X chromosome (Payer and Lee, 2008; Chen and Yen, 2019). *XIST* may recruit chromatin modification enzymes to participate in the regulation of chromatin structure, thereby leading to changes in the expression of oncogenic or anti-oncogene genes regulated by *XIST* (Cerase et al., 2015).

**FIGURE 2:**
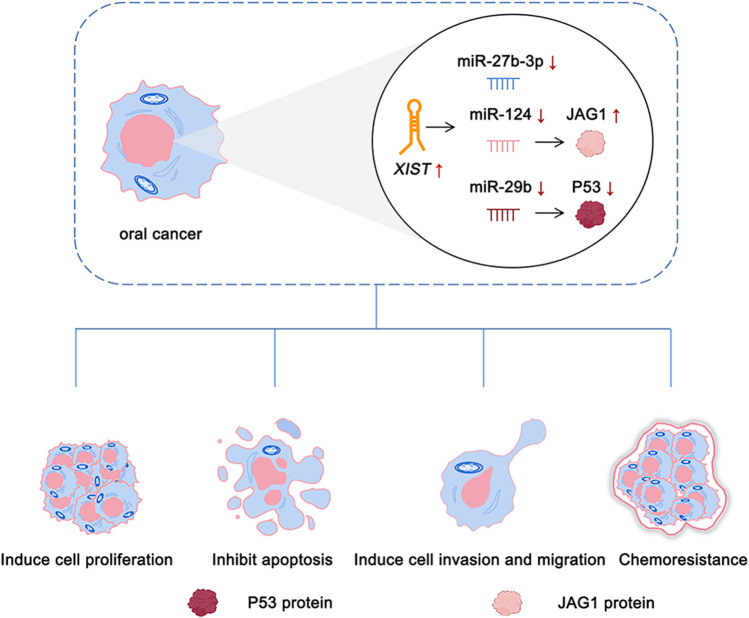
Role of *XIST* in oral cancer.

**FIGURE 3:**
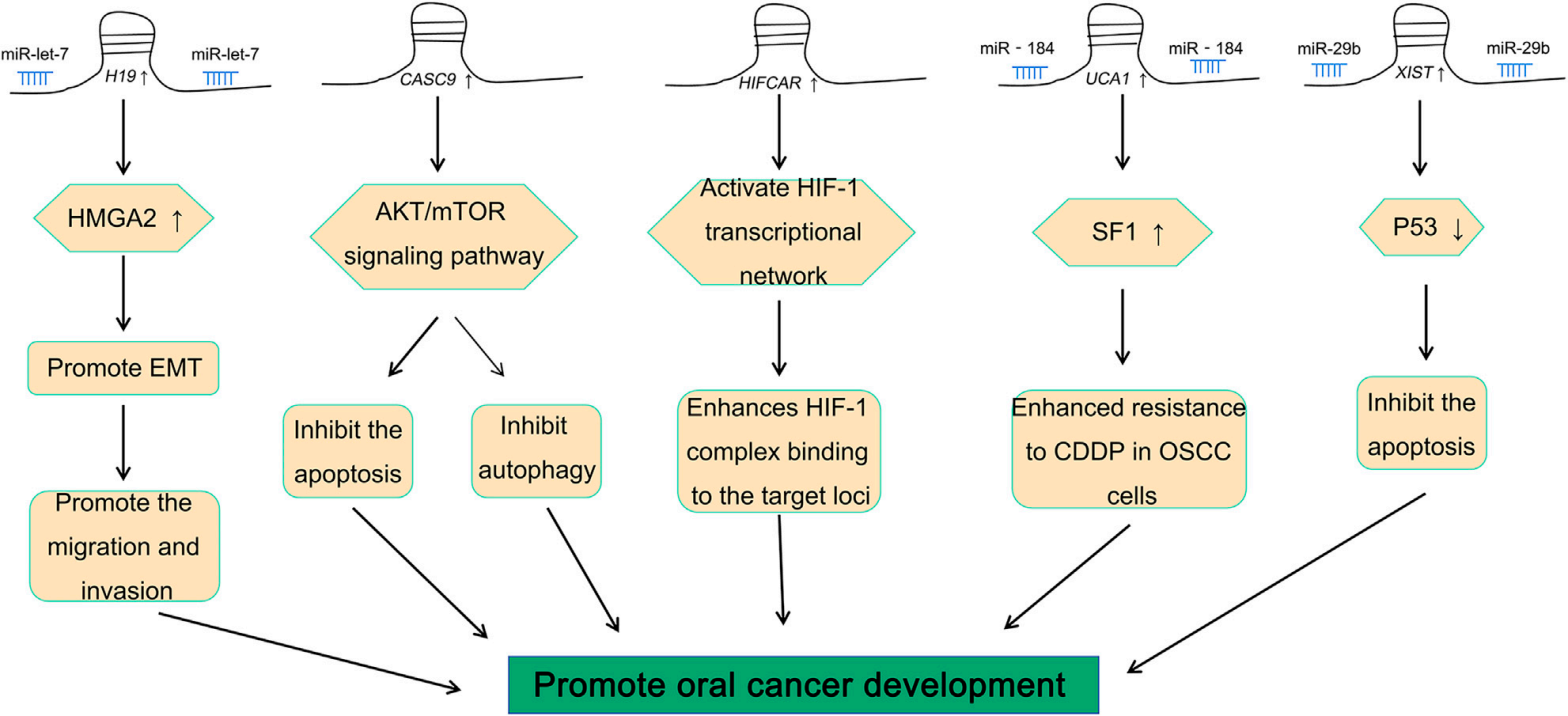
Role of *H19*, *CASC9*, *HIFCAR*, *UCA1*, and *XIST* in oral cancer.

There is an abnormal expression of lncRNA *XIST* in a variety of cancers, such as thyroid cancer (Liu et al., 2018), colorectal cancer (Chen and Shen, 2020), breast cancer (Soudyab et al., 2016), and oral cancer (Tao et al., 2021). A previous study by our group demonstrated that there was an abnormal expression of lncRNA *XIST* in TSCC (Tao et al., 2021). Knocking down the expression of *XIST* significantly inhibited the proliferation, migration, and invasion of TSCC and induced their apoptosis (Tao et al., 2021). The role of *XIST* in oral cancer has been investigated, and the potential mechanism has been elaborated (Table 1 and Figure 2). In addition, possible methods, such as RNA-seq and fluorescence *in situ* hybridization (FISH) assay, can be performed to determine the expression of *XIST* to provide a potential implication in oral cancer (Shiura and Abe, 2019; Tao et al., 2021; Wang et al., 2021). This suggested that many lncRNAs, especially *XIST*, are abnormally expressed in a variety of cancers, including oral cancer, and play a key role in their pathogenesis *via* the regulation of different pathways (Figure 3).

**TABLE 1:** Potential role of *XIST* in oral cancer.

Method	Result	Potential mechanism	Reference
RT-qPCR	*XIST* expression is upregulated in OSCC tissues, cell lines, and cisplatin-resistant oral cancer cell	LncRNA *XIST*/miR-27b-3p	Ma et al. (2020)
CCK-8	Promote proliferation and enhances resistance to cisplatin of OSCC		
RT-qPCR	The expression of *XIST* is upregulated in patients	LncRNA *XIST*	Gao et al. (2022)
CCK-8	Promote proliferation	/miR-124/JAG1	
Wound healing assay	Promote cell migration		
RT-qPCR	The expression of *XIST* is upregulated in patients		
CCK-8	Promote proliferation		
Flow cytometry	Inhibit apoptosis	LncRNA *XIST*/miR29b/P53	Tao et al. (2021)
Wound healing assay	Promote cell migration		
Transwell	Promote cell invasion		

## Regulatory mechanism of *XIST* and its expression pattern in cancer

**FIGURE 4:**
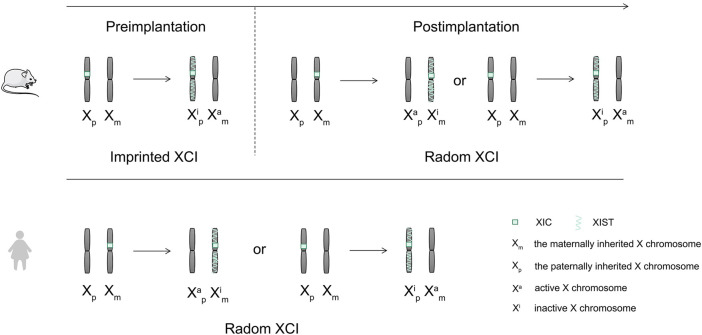
Mechanisms of X chromosome inactivation in mice and humans.

LncRNA *XIST* is the earliest discovered lncRNA (Loda and Heard, 2019). It mainly plays a role in regulating X chromosome inactivation in cells and can silence most genes on the inactivated X chromosome (Cerase et al., 2015). There are about 1,000 genes present on the X chromosome, which can cause a variety of diseases such as cancer and hemophilia (Lee and Bartolomei, 2013). Therefore, the role of *XIST* in X chromosome inactivation is important in mammals (Figure 4).

**FIGURE 5:**
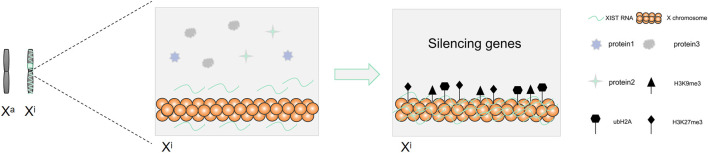
*XIST* mediates X chromosome inactivation and silences genes.

There are two X chromosomes that are present in the somatic cells of female mammals, while there is only one X chromosome present in the male mammalian cells (Wang et al., 2016). To maintain the balance of gene expression between female and male mammals in embryo development, female mammals achieve X chromosome dose compensation *via* X chromosome inactivity (Sahakyan et al., 2018). X chromosome inactivation (XCI) needs the activation of the X chromosome inactivation center (Dixon-McDougall and Brown, 2016). The X chromosome inactivation center (XIC) is located in the region from 100 to 500 kb on the X chromosome (Dixon-McDougall and Brown, 2016). The core of XIC is long non-coding RNA *XIST*, which is only expressed in the female inactive X chromosome (Gendrel and Heard, 2014). XCI includes two forms, the random XCI and the imprinted XCI (Sahakyan et al., 2018). Two forms of imprinted XCI and random XCI occur during the embryonic development of mice (Sahakyan et al., 2018). The imprinted XCI first occurs in the female mouse pre-embryonic stage, silencing the patrilineal X chromosome (Sahakyan et al., 2018; Chen and Yen, 2019). Subsequently, following blastocyst formation, the imprinted XCI remains unchanged in trophoblast ectoderm cells, while the cells in blastocyst ectoderm reactivate the paternal X chromosome, and random XCI occurred before and after implantation (Sahakyan et al., 2018). However, the dose compensation of the X chromosome is realized by random XCI in human (Sahakyan et al., 2018). *XIST* wraps around the X chromosome on which it sits, recruits heterochromatin factors, and silences gene expression (Figure 5). The expression of *XIST* only plays a role in the initiation of X chromosome inactivation. There is a need for other supporting factors to maintain the inactivation of the X chromosome (Fatica and Bozzoni, 2014).

During the process of X chromosome inactivation (XCI), the entire X chromosome is permanently silenced and transformed into Barr bodies (Dixon-McDougall and Brown, 2016). Studies have found that the expression of histone markers on the chromosome increased, such as H3K27me3, H3K9me3, and ubH2A, once *XIST* was enriched on the X chromosome (Mermoud et al., 2002; Plath et al., 2003; Fang et al., 2004; Brinkman et al., 2006; Dixon-McDougall and Brown, 2016). Meanwhile, the corresponding gene expression in the histone-rich region was silenced (Mermoud et al., 2002; Plath et al., 2003; Fang et al., 2004; Brinkman et al., 2006; Dixon-McDougall and Brown, 2016). *XIST* can recruit and bind Polycomb-repressive complex 2 (PRC2) to the transcriptional site of XIST gene, cause histone modification in this region, and mediate X chromosome inactivation (Wutz, 2011). This suggested that histone modification plays a key role in XCI. High DNA methylation also plays a role in stabilizing the inhibitory state of silent genes, thereby suggesting that DNA methylation plays an important role in maintaining the stability of XCI (Dixon-McDougall and Brown, 2016).

The link between XCI and cancer was proposed more than 50 years ago (Pageau et al., 2007). They found that there was a lack of Barr bodies in some breast cancer cells, thereby suggesting that the inactivation of the X chromosome may be related to cancer (Pageau et al., 2007). As a specific expression of lncRNA in female cells, a large number of studies have shown that *XIST* is closely related to the high incidence of cancer in women, such as breast cancer (Schouten et al., 2016), cervical cancer (Zhu et al., 2018), thyroid cancer (Liu et al., 2018), and ovarian cancer (Zuo et al., 2019). *XIST* acts as an oncogene to promote the development of cancer *via* multiple pathways. The abnormal expression of *XIST* has been observed in human oral cancer, and it has been found that *XIST* plays a major role in the pathogenesis of oral cancer (Tao et al., 2021). The aforementioned results suggested that the abnormal expression of *XIST* may lead to the abnormality of X chromosome inactivation, lead to a gene mutation on it, and then promote the occurrence of many diseases including cancer. These results suggested that *XIST* and XCI have a close connection with cancers including oral cancer.

## *XIST* acts as a sponge molecule

The “competitive endogenous RNA” (ceRNA) hypothesis was proposed in 2011 (Salmena et al., 2011). It was believed that the ceRNA mechanism formed a large-scale regulatory network in transcriptome and plays an important role in diseases such as cancer (Salmena et al., 2011). Major elements such as microRNAs (miRNAs), protein-coding genes, and lncRNAs are included in the mechanism of ceRNAs (Salmena et al., 2011). LncRNA can further regulate the expression of mRNA *via* competitive binding with microRNAs in a variety of cancers. Studies conducted in breast cancer have shown that lncRNA *BCRT1* acts as a molecular sponge of miR-1303, negatively regulates the expression of miR-1303, and upregulates the expression of PTBP3 *via* exocrine, which, in turn, promotes the development of breast cancer (Liang et al., 2020). In colorectal cancer, miR-181a-5p can be used as a target for lncRNA *CRNDE* to competitively combine with lncRNA *CRNDE* for promoting the growth of colorectal cancer (Han et al., 2017). *XIST* also can act as a sponge molecule for miRNA in cells. For instance, *XIST* upregulates Fus (fused in sarcoma) *via* competitive binding with miR-200a, which in turn acts as a ceRNA in the development of cervical cancer (Zhu et al., 2018). *XIST* and MET protein compete for the combination of miR-34a, which in turn leads to the progression of thyroid cancer (Liu et al., 2018). The aforementioned data indicate that *XIST* regulates the expression of target mRNA and target genes. A key role is played by lncRNA *XIST* in X chromosome inactivation. It also acts as a sponge molecule of microRNAs to participate in the ceRNA regulatory network in the development of oral cancer. Previous research by our group suggested that *XIST* is abnormally expressed in TSCC and acts as a sponge molecule to compete with miR-29b to stimulate the growth of TSCC (Tao et al., 2021).

*XIST* regulates the expression of miRNAs *via* the ceRNA mechanism and downstream signaling pathway to impact the growth of cells. Studies have found that *XIST* can act as the sponge molecule of miR-34a to participate in ceRNAs in thyroid carcinoma cells and further inhibit the growth of thyroid cancer cells *via* its downstream signal pathway MET (hepatocyte growth factor receptor)-PI3K (phosphoinositide 3-kinase)-AKT (α-serine/threonine-protein kinase) signal pathway (Liu et al., 2018). Abnormal glucose metabolism is very important for the progression of cancer. It has been previously demonstrated that lncRNA *XIST* can control the growth and glucose metabolism in glioblastoma cells *via* the insulin receptor substrate 1 (IRS1)/the phosphoinositide 3-kinase (PI3K)/protein kinase B(Akt) pathway (Cheng et al., 2020). To summarize, studying the mechanism and signal pathway of *XIST* in other cancer cells may provide a theoretical foundation for the future study of *XIST* in oral cancer. *XIST*, as a molecular sponge, plays important role in oral cancer by regulating miRNA and competitively binding with target mRNA.

## The role of *XIST* in other cancers

As mentioned previously, a link between the X chromosome and cancer has been found (Pageau et al., 2007). *XIST*, which is the core of XCI, plays a key role in many cancers (Zhang et al., 2018b; Liu et al., 2019; Li et al., 2020b; Liu et al., 2020b). High expression of *XIST* can be used to identify BRCA1-like breast cancer and has a worse prognosis than BRCA1-like breast cancer patients with low expression of *XIST* (Schouten et al., 2016). *XIST* can also upregulate the expression of the Polycomb group protein RING1 mRNA *via* competitive binding with miR-744 and further promote the occurrence and development of non-small cell lung cancer (NSCLC) *via* the Wnt (Wingless/Integrated)/β-catenin signal pathway (Wang et al., 2019b).

The tumor microenvironment plays a key role in the occurrence and development of cancer (Chanmee et al., 2014; Zhao et al., 2021). Previous studies have reported that tumor-associated macrophages (TAMs) in the tumor microenvironment are closely related to tumor proliferation, migration, and angiogenesis (Zhao et al., 2021). *XIST* can regulate the polarization of tumor-associated macrophages through miR-101-3p and then promote the proliferation and migration of breast and ovarian cancer cells (Zhao et al., 2021). It also promotes the development of colon cancer *via* the miR-34a-mediated Wnt/β-catenin signaling pathway (Sun et al., 2018). Thus, the expression of *XIST* is upregulated in a variety of cancers that promotes the occurrence and development of cancer *via* different mechanisms. To summarize, *XIST* is abnormally expressed in a variety of cancers, including oral cancer. It can affect the occurrence and development of cancer by inhibiting apoptosis and promoting proliferation, invasion, and migration of cancer cells.

## Discussion

Oral cancer is a common malignant tumor of the oral and maxillofacial region. It has a surgical injury and a high fatality rate. Multiple factors are involved in the pathogenesis of oral cancer (Gharat et al., 2016). External factors including smoking (Huang et al., 2019), drinking (Varoni et al., 2015), and chewing betel nut (Su et al., 2020), and internal factors like gene mutation (Ali et al., 2017), the human papillomavirus (HPV) (Jiang and Dong, 2017), and abnormal gene expression also play a major role in the development of oral cancer. As a class of gene regulatory factors, lncRNA plays an important role in oral cancer cells. It can be used as a gene target in the study of oral cancer gene therapy. The expression pattern of lncRNA *XIST* is particularly important in oral carcinoma. *XIST* binds competitively with miRNA and affects apoptosis, proliferation, cycle, and migration and also influences other related genes of cancer cells, such as cyclinD1 (Wang et al., 2019b), p53 (Hu et al., 2019), and E-cadherin (Shi et al., 2020).

Some epigenetic modifications are closely related to a variety of human diseases including cancer (Villanueva et al., 2015). *XIST* is regulated by some epigenetic modifications, such as DNA methylation and M^6^A modification. For example, the expression of M^6^A “writer” protein methyltransferase-like 14 (METTL14) is downregulated in colorectal cancer, and it has been found that *XIST* is downregulated by METTL14 in a YTHDF2-dependent way, which is responsible for inhibiting the growth of colorectal cancer (Yang et al., 2020).

Studies found that targeted regulation of the expression of *XIST* in oral cancer, and some other cancers can inhibit the occurrence and development of tumors (Li et al., 2020b; Shi et al., 2020; Tao et al., 2021). The expression of lncRNA *XIST* is increased in non-small cell lung cancer (Wang et al., 2019b). A decrease in the expression level of *XIST* in lung cancer cells significantly reduced their proliferation and invasion (Wang et al., 2019b). In glioblastoma cells, knocking down the expression of *XIST* also significantly reduced the glucose uptake of glioblastoma cells and helped to inhibit tumor growth (Cheng et al., 2020). Similarly, studies have shown that knocking down the expression level of *XIST* can significantly inhibit the proliferation and promote the apoptosis of oral cancer cells (Tao et al., 2021). The abnormal expression of *XIST* was shown in oral cancer. Moreover, knockdown expression of *XIST* has the ability to induce apoptosis and inhibit cell growth, migration, and invasion (Ma et al., 2020; Tao et al., 2021; Gao et al., 2022). Moreover, TCGA (http://ualcan.path.uab.edu/analysis.html) database showed that *XIST* was closely associated with the rainfall of HNSCC, and patients with higher *XIST* expression levels had a worse prognosis.

Some drugs can inhibit the expression of *XIST* and induce cell apoptosis. *Platycodon* *grandiflorum* saponin D (PD) is a saponin extracted from *Platycodon grandiflorum* (Chen et al., 2020). Studies have demonstrated that PD reduced the expression of *XIST* and inhibited the growth of bladder cancer cells (Chen et al., 2020). Research carried out by our group has shown that cucurbitacin B effectively inhibited the proliferation of oral cancer and significantly reduced the expression of *XIST* in oral cancer cells (Tao et al., 2021). *XIST* can regulate the progression of oral cancer *via* multiple mechanisms. This suggested that *XIST* can potentially be an important target for the diagnosis and treatment of oral cancer.

## Conclusion

Many different lncRNAs have been found in the human genome, which are involved in various biological processes of cells. They are important regulatory factors in these biological processes. *XIST* is an important initiator of X chromosome inactivation and a gene regulator like other lncRNAs in many cancers, including oral cancer. An abnormal expression of *XIST* is seen in a variety of cancers, including oral cancer. *XIST* promotes proliferation, invasion, and migration, and inhibits apoptosis. Moreover, *XIST* can increase the drug resistance of cancer cells; this may seriously increase the difficulty of chemotherapy treatment for oral cancer. This indicated that the abnormal expression of *XIST* is closely related to the occurrence, development, and treatment of oral cancer. Therefore, *XIST* can be a potential new target for the treatment of oral cancer.
